# The Anatomy of the Human Brain
*By Holmes Coote, Demonstrator of Anatomy at St. Bartholomew's Hospital.


**Published:** 1850-04-01

**Authors:** 


### Art. IV.—THE ANATOMY OF THE HUMAN BRAIN.*

The advance made within the few last years towards a more accurate and scientific knowledge of the nervous centres is due to microscopical investigations, and to the study of comparative anatomy; the former having brought to light the minute structure of the nerve tubes, of the vesicular matter with which they are connected, and the arrangement of the capillary vessels which minister to their growth and repair; the latter having shown the signification of a nervous centre, the relative value of its different segments, and of the parts by which it is surrounded. Had anatomists followed the precepts and example of John Hunter, and traced the nervous system in its gradations, step by step, from the lower to the higher classes of the animal kingdom, we should have been spared those long discussions to disprove that the spinal chord is made up of an assemblage of nerve tubes, which pass in an unbroken line from the periphery to the brain; or those figurative expressions, so productive of error, which pronounced the spinal chord to be a prolongation of the brain, or the brain to be an efflorescence of the spinal chord. Both the brain and chord are developed contemporaneously in early fœtal life as a single hollow membranous cylinder, of which the anterior extremity, more dilated than the rest, becomes eventually the encephalon. The deposition of nervous matter commences upon the under or abdominal wall of the cylinder; small granular and nucleated bodies (*cytoblasts*) are arranged in a double linear series; the fissure between them, separating the right from the left half, being still traceable in the adult spinal chord as the posterior median fissure. The further accumulation of nervous matter within the membranous cylinder, and the coalescence of the two halves along its dorsal aspect, leads to the formation of a central canal, which is persistent in fish, but obliterated in the mammalian class; this canal leads into the anterior encephalic or cranial dilatation, the brain vesicle, which soon becomes constricted into three smaller vesicles, the rudiments of the cerebellum, the mesencephalon, and the cerebral hemispheres. A fourth segment, the olfactory (rhinencephalic), is subsequently added. About the third month of fœtal life in the human embryo, the cerebral hemispheres rapidly increase in size, and extend backwards so as to overlap the other cranial vesicles; but

* By Holmes Coote, Demonstrator of Anatomy at St. Bartholomew's Hospital.

the median canal, obliterated in the chord, is persistent in the brain; it leads from the mesencephalon, under the nates and testes, to the vesicle of the cerebellum, and is known as the iter e tertio ad quartum ventriculum. The communication between the mesencephalic and hemispheric vesicles, though masked, and divided by the development of the fornix, is still recognised in the adult brain as the foramina of Monro. The gradual perfection of the brain is marked by the separation of the white and grey nervous matter; by the introduction of longitudinal and transverse commissures bringing into connexion its different parts; and by the development of those superficial inequalities known as convolutions, which, however tortuous and subdivided, are yet under the control of a fixed law, by which their number and course are regulated. The incomplete separation of the white and grey matter, errors in their due proportion, or the imperfect development of commissures, are always associated with impairment of function. Excess of fluid in the cranial vesicles leads to their rupture and collapse: from this cause is produced the anencephalous fœtus, in which we notice a membranous sac resting upon a basis cranii, but surrounded by a skull-cap, so imperfectly constructed, that no small skill is requisite to designate its component pieces. But in the anencephalous fœtus the skull is still composed of its proper number of bones, however much deformed or twisted they may be.* Some idea of the period of collapse of the brain vesicles may be entertained from the study of the deformed bones by which they are surrounded. It has been correctly remarked by Hyrtl, that inasmuch as the proportional development of the organs which preside over vegetative life, and of those which are connected with the psychical manifestations, is regulated by the period of existence, so the brain is the less fitted for its higher functions the earlier the arrest of development, and the more the organ retains its embryonic simplicity. Indeed, in these monstrosities the parts and organs connected with vegetative life seem to have acquired undue activity in growth; the upper, the lower jaws, and the os hyoides, are absolutely larger than the same bones in the well-formed fœtal skull of equal age: but the bones at the base of the skull become coalesced and blended together, so that their separation cannot be effected. The arrest in the developments of the parts, both external and internal, is in obedience to one and the same law.

Microscopical Anatomy.—The cerebro-spinal centre is com-

* In the Museum of St. Bartholemew's Hospital, there is a preparation of the skull of an anencephalous fœtus, disarticulated; the bones properly marked and numbered.

posed of vesicular and of fibrous nervous matter; of fatty and granular matter; of pigment, epithelium, and bloodvessels.

Vesicular Nervous Matter.—Vesicular nervous matter, which preponderates greatly in the grey substance, whether spread over a surface as in the cerebral convolutions, or accumulated in central masses as in the spinal chord, consists of cells, containing usually one nucleus, but sometimes two, in which may be seen nucleoli. The cells are round, oval, or angular, and are contained in a sheath or capsule of very delicate membrane: when angular, one or more of the angles may be prolonged into a tail with rounded or with sharp extremity; some of the cells are fusiform or caudate. Their dimensions, says Hannover, varies from the size of a globule of human blood to six or seven times that of a globule of frog's blood. The largest are those in the grey matter of the spinal chord. Next follow, in the order of size, those of the cerebellum, cerebral hemispheres, and olfactory bulbs. The small cells abound most in the cerebellum and the tubercula quadrigemina. Vesicles may be found in every part of the nervous system where the substance is not perfectly white.

Fibrous Nervous Matter.—Nerve fibres are cylindrical tubes of different diameters; they are composed of a fibrous sheath (neurilemma) and an homogeneous, semi-transparent, easily coagulating fluid. Hannover,* who has particularly examined the minute structure of nerve tubes, describes, in the large fibres taken from the floor of the fourth ventricle, two double outlines on either side, of which the two exterior belong to the cellular sheath, the two interior to the marrow. Within the sheath and the marrow is a cylindrical axis, which pursues exactly the course of the nerve fibres. There are many small nerve tubes amongst those of larger size: their structure is supposed to be in every respect similar, although, from their delicacy, the outlines are not clearly marked. The large fibres abound in the spinal chord, the small fibres in the grey substance of the brain, and in the retina. The fibres of the former part are more elastic than those in the latter. From mechanical or chemical causes, the fibres, which, in the fresh state, are rectilinear and transparent, become varicose, the swelling affecting sometimes only one side, sometimes both: and upon examination it will be found that this appearance depends upon the unequal accumulation of the fluid which surrounds the central cylindrical axis in different parts of the fibrous sheath.

Origin of the Nerve-Fibres.—The nerve-fibres have been dis-

* Recherches Microscopiques sur le Système Nerveux. 1844.

tinctly traced to arise from the nerve vesicles, with the outer wall of which, and not with the nucleus, they are continuous. As a rule, a vesicle gives origin to a single nerve-fibre; it is less common to find two arising from the same source. Care must be taken, in investigating these points, not to confound with true nerve-fibres the caudate prolongations terminating in blunt extremities, noticed in the triangular vesicles of the grey nervous matter. There are other vesicles from whence no nerve-fibres arise; and as the fibres are by far more numerous than the vesicles, it has been conjectured by Hannover, that upon the perfect development of the fibre, the union between it and the vesicle ceases. The fibres pass from the surface of the brain towards its base; then traversing the different accumulations of grey matter in the interior, become continuous with the white fibres of the chord; but many escape to form the cerebral nerves, which emerge from the cranial foramina. In the chord, nerve-fibres arise from the central grey nucleus, and curve outwards after descending, for a short distance, to constitute the spinal nerves. These remarks apply to the fine as well as to the large nerve-fibres, although the origin of the former is less directly capable of proof by ocular demonstration. The presence of the fine fibres in nerves, and especially in the branches of the sympathetic, was noticed first by Remak, who pointed out their close resemblance with the smaller and more delicate fibres in the cerebro-spinal axis; and there is now little doubt of the continuity of the peripheral and the central fibres: the latter, constituting an integral part of a nervous centre, are surrounded by a more delicate cellular sheath, and are consequently more prone to varicose dilatations, but they suddenly become larger upon leaving their point of origin, and are bound together by a coarser tissue, to form those white chords which we commonly recognise as nerves.

THE CEREBRO-SPINAL AXIS may be regarded as an assemblage of ganglia, corresponding in number with the vertebral segments of the spinal column, and giving origin, each in its turn, to a pair of nerves. And to this definition, the ganglia constituting the brain or encephalon offer no exception. Since Oken's discovery, the skull has been recognised as part of the vertebral column, composed of four segments, of which three are well formed; and the fourth, the most anterior, is cartilaginous in cold-blooded animals, and obscured by the development of an organ of special sense, the olfactory, in the higher mammalia. The cranial vertebræ are as follows:* the

* This description comprises only the upper or neural half of the cranial vertebræ; the lower or hæmal half, being unconnected with the nervous centres, is not mentioned.

occipital, formed by the occipital bone; the parietal, formed by the posterior part, and great alæ of the sphenoid, and by the parietal bones; the frontal, formed by the anterior part, and lesser alæ of the sphenoid, and by the frontal bones; and the nasal, formed by the vomer, with its ossa plana and perpendicular lamella, and the nasal bones. The occipital, parietal, and frontal vertebræ correspond with the three primary constrictions of the embryonic brain vesicle; the nasal, with the subsequently developed olfactory bulbs. The assemblage of pieces constituting the human temporal bone (viz., the squamous, mastoid, typanic, stylo-hyal, and petrosal bones) is intercalated in the skull to occupy the space between the occipital and parietal vertebræ, caused by the enormous backward development of the cerebral hemispheres. The vertebrate character of the rings of bone surrounding the simpler and more typical encephalon of some of the cold-blooded vertebrata—*e. g.*, the turtle—can be easily recognised. Like as each ganglion or segment of the chord gives off its pair of nerves, corresponding with the vertebra which surrounds it, so in the same way does the chain of cerebral ganglia; the two first pair containing both motor and sensitive filaments; the two last being nerves of special sense. It is true that several of these chords divide within the cranium, and their branches emerge by separate foramina, but in these cases both the origin and distribution indicate their affinity with spinal nerves. The occipital segment (epencephalon) gives off the hypoglossal or motor nerve of the tongue, and the glosso-pharyngeal and pneumogastric nerves, which bestow sensation upon the fauces, œsophagus, stomach, air-passages, and lungs. The spinal accessory nerve, though receiving filaments as low as the fifth cervical vertebra, is connected with the medulla oblongata, or upper extremity of the chord, in the close neighbourhood of the pneumogastric. The motor and sensitive nerves of the parietal or second segment (mesencephalon) comprise the three divisions of the fifth, and the third, fourth, sixth, and facial nerves. The ophthalmic and the motor nerves of the muscles of the eye pass into the orbit: the superior maxillary, with the facial nerve, supplies the greater part of the face; the inferior maxillary, containing both motor and sensitive filaments, terminates in the organs of mastication. Through the foramina of the frontal vertebra, which contains the prosencephalon, pass the optic nerves; through those of the nasal vertebra, surrounding the rhinencephalon, the olfactory; both supplying organs of special sense. It would be impossible in the limits of this paper to trace the nerves of communication connecting these several chords. As illustrations, I would mention the great petrosal nerve, extending between the superior maxillary and the facial nerves; and the numerous filaments passing between the ophthalmic and motor nerves of the orbit; or the intimate connexion between the hypoglossal and the nerves composing the eighth pair. In the study of the cerebro-spinal axis, the anatomist should not limit his views to the information afforded by the human subject: he should trace the modifications of encephalic development through the different orders of the vertebrate sub-kingdom; he should notice the varying proportions of the primary cranial ganglia, their gradual diminution in size, and eventual disappearance; until, as in the Lancelet (branchiostoma) there remains but the faintest trace of a brain, or of the organs with which it is associated. The neural axis of the lancelet, composed of vesicular matter (nucleated cells), terminates by an obtuse end at its cranial extremity, and gives off but two nerves, the trigeminal and the optic; the nerves corresponding with the hypoglossal, and the three divisions of the eighth pair belong to the system of spinal nerves. But in all other fishes, the cranial extremity of the chord supports ganglionic masses, arranged in linear series, and giving off the vagal and trigeminal nerves, and the nerves of special sense.

Membranes of the Cerebro-spinal Axis.—The membranes of the neural axis are three: the dura mater, arachnoid membrane, and pia mater.

The dura mater, composed of white inelastic fibrous tissue, supports the spinal chord; and, within the cranium, becomes closely applied to the inner surface of the bones, forming an internal periosteum. It is the opinion of Dr. Sharpey that the cranial bones are developed, not from temporary cartilage, but from the fibrous tissue of the dura mater.

It is certain that one fails in detecting cartilage in the stages of ossification of the fœtal frontal or parietal bones; and the intimate vascular connexion between the dura mater and the pericranium is maintained through life, in spite of the deposit of earthy matter by which they are separated. The membranous partitions (falx cerebri, tentorium cerebelli, &c.) are ossified in some animals, as the cat, dog, &c.; but this conversion of membrane into bone cannot be connected with the firm support of the brain in the active movements of the animal; for it is found in the ornithorhynchus, an animal formed for burrowing in the soft mud of the Australian rivers. The adhesion of the dura mater to the skull is very firm in cases of chronic inflammation and cerebral affections: bony growths from the interior of the skull generally produce a thinning of the membrane, which is rendered transparent.

The arachnoid membrane is the serous membrane; it offers points of interesting inquiry both in its healthy and morbid state. It is described as lining the dura mater, and being thence reflected upon the outer surface of the pia mater. But it is a mistake to suppose that a distinct vascular membrane, coated by epithelium, is spread over the inner surface of the dura mater; the so-called parietal arachnoid consists only of a layer of epithelium; its vessels are the vessels of the dura mater, and morbid appearances observed upon it, result from inflammatory changes in the same membrane. The false or adventitious membrane, formed occasionally upon the inner surface of the parietal arachnoid (and which has been referred, by some authors, to the organization of extravasated blood), should be regarded, not as a disease of the arachnoid membrane, but as a disease of the dura mater; and this will explain why adhesions so rarely form between the opposed surfaces of the arachnoid. It does not by any means follow that the pia mater and visceral arachnoid should be inflamed, because of inflammation of the dura mater.

These adventitious membranes, so rarely seen in ordinary hospital practice, but not by any means uncommon in the bodies of those who die in lunatic asylums, result from inflammation, which, during life, manifests itself by interfering with and disturbing the proper functions of the brain. The class of persons in whom such morbid changes are found after death, are noticed by their friends for months, perhaps for years, to be irritable and easily excited, or to become dull and morose; delirium often supervenes, and frequently paralysis, which, in the cases examined by myself, has depended upon softening of some part of the surface of the encephalon. I have heard it said that these false membranes are formed unconnected with any change of structure in the dura mater; that statement is totally at variance with my dissections. The dura mater will be found to be either extremely vascular, or most firmly adherent to the cranial bones; in short, it presents the usual indications of previous inflammation. The visceral arachnoid, connected with the pia mater, has its own vascular layer; the morbid changes most commonly seen in it are interstitial deposition, thickening, and opacity. Does the arachnoid membrane enter the cavity of the ventricles by the fissure of Bichat, as usually described? I believe not. In the first place, it is not possible to introduce a probe, however fine, under the corpus callosum into the ventricles: in the second place, the ventricles are lined by ciliated epithelium, and not by the tesselated epithelium of the arachnoid: and in the third place, it never happens that fluid accumulated in large quantity in the ventricles, makes its escape from the cavity when, in the examination of the head, the sac of the arachnoid is opened.

The pia mater, or the vascular membrane of the neural axis, is composed of a network of small vessels, which closely invests the surface of both brain and chord, following the inflexions and convolutions in every part, and lining those internal surfaces known as the walls of the ventricles. The blood-vessels form minute polygonal spaces; the arteries, which pass into the nervous substance, are given off at right-angles to the trunks, which form the network. In the spinal chord, the greatest number of vessels is found along the course of the anterior and posterior median fissures, where they dip down to ramify in the grey nucleus. The pia mater of the brain, which covers the grey matter of the convolutions, is more vascular than that of the chord. Infiltration of fluid converts the membrane into a loose, spongy mass, which may be pulled from between the convolutions. In old age, when the convolutions are shrunken and atrophied, the fluid in the layers of the pia mater occupies the cerebral interspaces, which may attain considerable size.

The Spinal Chord.—The spinal chord extends from the foramen magnum to the first or second lumbar vertebra, from whence it is attached to the extremity of the canal by a fibrous chord, named filum terminale. It lies nearer the bodies than the arches of the vertebræ, and is separated from them by a venous plexus, external to the dura mater. In form, it is a cylinder, compressed from before backwards; but its diameter is not in all parts the same. Both in the cervical region, from the fifth cervical vertebra to the second dorsal, and in the lumbar region, from the eleventh dorsal to the extremity of the chord, there is a marked increase in size, from the accumulation of grey matter. These swellings correspond with the origins of the large nervous chords, which supply the upper and the lower extremities. The chord is composed of two symmetrical halves, separated by an anterior and a posterior median fissure, in which dip the vessels of the pia mater: both fissures are lined by white matter, and it is an error to suppose that the posterior reaches the central grey matter. The white matter at the floor of the fissures serves as a commissure between the two halves of the chord. There are also noticed in either half of the chord, two fainter marked collateral lines, which correspond with the anterior and posterior roots of the spinal nerves. Beside the above, other linear impressions have been described, but their existence in the fresh chord is still doubtful, and they are destitute of any physiological interest. Upon making a transverse section of the spinal chord, it will be found that the central grey matter, presenting some varieties of outline in the different regions of the spine, appears as two crescents, with the concavities directed outwards, and united by a transverse band of grey matter, which crosses from one half of the chord to the other, between the two median fissures. The anterior cornu does not reach the surface of the chord, but terminates in the white matter by a blunt extremity. The posterior is continuous with the posterior roots of the spinal nerves. Anatomists have chosen, for convenience of description, to divide the white structure of the chord into tracts: all that part between the posterior cornu and the posterior median fissure is the posterior tract or column; all that between the posterior cornu and the anterior median fissure, passing over the blunt extremity of the anterior cornu, is the antero-lateral tract or column; but it must be remembered that these tracts are not divided by any well-marked natural fissures. In the lower vertebrata, the proportion of white to grey matter is much greater than in man; the grey matter, too, loses its characteristic colour so as to be with difficulty distinguished by the naked eye: in fish, its presence was for some time totally denied.

Thirty-one pairs of nerves, exclusive of the nervus accessorius, take their origin from the spinal chord. Every nerve arises by two roots, separated by a process of pia mater, the ligamentum dentatum: the posterior root passes into the posterior cornu of grey matter; the anterior, intimately blended with the external white fibrous structure of the chord, is also continuous by means of some of its fibres with the anterior cornu. Ganglia are formed upon all the posterior roots, before the union of the two to constitute a perfect spinal nerve. Amongst the few facts worthy of credence, relative to the functions of different parts of the nervous system, there is none supported by better evidence than that of the anterior roots of the spinal nerves being motor, and the posterior, sensitive; but it is not yet so clear that motion and sensation can be referred to particular portions of the chord itself; we are not justified, from experiments upon the roots of nerves, in assigning special functions to particular tracts of white matter. Within the cranium, the chord becomes again enlarged; from the lower border of the pons Varolii, to the spot known as the decussation of the pyramids, it is called medulla oblongata; the white fibres become gathered together into bands, and the posterior tracts separate so as to leave exposed the central grey nucleus here highly developed. We notice two flat bands, one on either side of the anterior median fissure, the anterior pyramids; external to them two oval bodies, the corpora olivaria; next, two round chord-like bands, corpora restiformia; within these the posterior pyramids; and finally, by the remains of the posterior median fissure, two faintly-marked chords, the fasciculi teretes. Although it is doubtless desirable that these superficial markings should be properly indicated by names, care must be taken not to attach to them an importance due to the deeper fibres. That typical constancy which we observe throughout the vertebrate sub-kingdom, in the primary divisions of the nervous centres, is not adhered to in these superficial linear markings of the medulla oblongata. Let any one examine the posterior surface of the chord in the leopard; the columns are too numerous to be named otherwise than by numbers. I apprehend one object gained by the formation of these fibrous bands is the more ready passage of the white fibres into the masses of grey vesicular matter which, supported by the cranial prolongation of the chord, constitute the encephalon. We will attempt to follow the fibres into each of the four primary encephalic ganglia, premising that the term medulla oblongata is artificial, and designates no true natural division of parts.

The restiform bodies, diverging from one another, and leaving exposed the grey centre of the chord, curve upwards, and pass into the accumulation of grey matter, constituting the cerebellum. From the under surface of the cerebellum, two bands of white fibres, the processus e cerebello ad testes are continued to the next pair of cranial ganglia. Between the processus e cerebello ad testes, and the restiform bodies, are the fibres of the pons Varolii; and these three constitute the crus cerebelli. Now, a cerebellum is seen in its simplest condition in the cold-blooded vertebrata, and in birds; we there find it composed of that part of the organ, known in man as the superior and inferior vermiform processes; or that central disc to which the two lateral lobes of the human cerebellum are appendages. It is wrong, then, to speak of the vermiform processes as commissures; the terms are ill-chosen, as they are applied to the fundamental part of the organ. Observation seems to warrant our regarding the cerebellum as an organ presiding over combined muscular action. It is singular that, in its description, white fibres should be traced into it from the posterior columns of the chord only; for the central white fibres of the crura cerebelli, connected with the pons Varolii, are described as commissural, and passing from one lateral lobe to the other. I think that this account is incorrect. The pons Varolii contains white and grey matter, receiving and giving origin to nerve fibres, and therefore cannot be rightly regarded as a mere commissure. The superficial fibres are commissural; but the deeper, and by far the more numerous, spring from the remains of the anterior median fissure, from the anterior columns, and turning outwards to the right and to the left, pass into the cerebellum in the same manner as do the fibres constituting the restiform bodies. In the rabbit's brain, which presents only the rudiments of the lateral lobes of the cerebellum and the pons Varolii, I have nevertheless succeeded in tracing from the anterior columns of the chord, those fibres forming the middle part of the crura cerebelli.

About an inch and a half below the pons may be seen that interchange of fibres in the anterior median fissure, known as the decussation of the pyramids. Too much importance has been attached to this spot; the number of nerve fibres which pass from right to left, and *vice versâ*, is inconsiderable, and the commissure most probably affects only that segment of the chord in which it is found. Between the decussation of the pyramids and the pons, commissural fibres may be seen, upon the separation of the two halves of the chord, to pass from the posterior to the anterior columns: some of these, emerging from the anterior median fissure, and curving under the olivary bodies, have been called the arciform fibres. The same decussation exists in the central line of that complicated part of the encephalon, the pons Varolii; although, from the accumulation of grey matter, the anterior median fissure is obliterated, the passage of white fibres from side to side may be distinctly made out: some of them pass into the crura cerebelli.

The space between the posterior surface of the medulla oblongata and the under surface of the cerebellum is called the fourth ventricle: its floor is composed of the grey matter of the chord left exposed by the divergence of the restiform bodies. In it are seen elevations connected with the roots of the pneumogastric and glosso-pharyngeal nerves, and transverse white fibres (the feather of the Calamus Scriptorius), which are regarded as the origin of the acoustic nerves. The lower opening of the fourth ventricle is shut up by pia mater and arachnoid membrane; the upper opening is continued into the space between the two optic thalami or the third ventricle, by a canal, the remains of the central canal of the cerebro-spinal axis, and called the iter e tertio ad quartum ventriculum. In the crus cerebelli is a zigzag line of yellowish grey matter, the corpus dentatum. The smooth tongue-shaped cerebellum of osseous fishes, which move with but little effort in an atmosphere of sufficient density to support them, is superseded, in the predacious cartilaginous sharks, by a cerebellum, of irregular surface, and marked by transverse foldings. So also in birds which soar by muscular exertion in the rarer strata of the atmosphere, the cerebellum is plicated, and the division between white and grey matter is distinct. The size of the cerebellum seems to depend less upon the required amount of muscular strength than upon the necessity of rapid and varied muscular combinations. Man, inferior to the lion in point of strength, is immeasurably his superior in the extent, accuracy, and delicacy of his movements.

The second cranial segment, or ganglion (mesencephalic), comprehends the nates and testes, and that portion of the pons Varolii connected to them. The mesencephalon is surrounded by a ring of bone, which, though greatly expanded in man, retains in the lower vertebrata, its typical relations, and is formed by the posterior half of the sphenoid bone, and its greater alæ, (basi and ali-sphenoids,) and by the parietal bones. If the corpora olivaria be cut across, we find, in the interior, a corpus dentatum, formed of a zigzag line of yellowish-grey matter, as in the crus cerebelli. The white fibres, both surrounding the corpus dentatum, and springing from its interior, pass into the pons, where they are joined by fresh fibres. They unite to form a band, which divides into two: one passes to the nates and testes; the other, to the crura cerebri.

The nates and testes, composed of white and grey matter, are two pair of smooth, roundish bodies, which retain much more than the cerebral hemispheres, or the cerebellum, that simplicity of structure seen in the cold-blooded animals. Obscured from view by the great backward development of the hemispheres, they are not seen until the contiguous parts are somewhat separated; and their true importance might be overlooked, were it not for the information afforded by comparative anatomy. The corpora bigemina in fish, the homologues of the nates (for the testes do not exist), far exceed in size the other cerebral ganglia, and present a greater complexity of structure than is found in the human brain. Indeed, Cuvier regarded the hollow lobes, as they were then called, as the hemispheres. The addition of an inferior pair is first seen in the implacental mammalia; the relative size of the two depends upon causes not perfectly understood. The nates seem connected with the origin of the optic nerves, which wind round the crura cerebri, to be attached to the corpora geniculata upon the optic thalami. The testes, resting upon the valve of Vieussens in front of the origin of the fourth cerebral nerve, are relatively largest in the active carnivorous mammalia.

The nerves connected with the mesencephalon are numerous, and divide within their cranial vertebra, before passing through different foramina to their destinations. The nerves of sensation are represented by the divisions from the Gasserian ganglion of the nervus trigeminus. The motor nerves include all the motor nerves which accompany their branches. The third, the fourth, the sixth, the portio dura, and the motor root, which accompanies the inferior maxillary nerve, may be regarded as so many divisions of an anterior motor root of a spinal nerve.

The ring of bone surrounding the cerebral hemispheres is formed by the anterior part of the body of the sphenoid bone, the processes of Ingrassias and the frontal bone. It contains the largest segment of the encephalon. The two round chords, known as the crura cerebri, receive fibres from the anterior pyramids, from the olivary tracts, and from the grey matter of the pons. Doubtless, also, fibres pass in the opposite direction, arising from the accumulation of grey matter within the hemispheres. The separation of the white and grey structures of the brain in distinct layers, imperfect even in the rodentia, is complete in man, who, in his intellectual faculties, is so greatly superior to all other animated beings. The crura cerebri can be traced into three accumulations of grey matter—first, into the optic thalami; secondly, into the corpora striata; and thirdly, into the grey matter of the convolutions. The hemispheres, thus formed, are united by a transverse layer of white fibres, the corpus callosum; and the vault between them, divided into spaces by the development of intercerebral commissures, constitutes the lateral and the third ventricles. All persons are familiar with the way in which the grey matter of the surface of the hemispheres is spread out in wavy lines, coating the central white matter, and forming the convolutions; and the course and number of these convolutions is not a matter of chance, nor do they adapt themselves to the inner surface of the skull; they are under the control of some law, which as yet is but imperfectly understood. In the lower vertebrata, and in most birds, there is no trace of cerebral convolutions; in the implacental mammalia, and in rodents, the superficial markings are faint. In the feline carnivora, the convolutions, commonly four in number in either hemisphere, extend longitudinally from before backwards, parallel to the median fissure, and to one another. In the herbivora, *e. g.*, the sheep, they are oblique, converging towards the median fissure from behind, forwards. In man they are so numerous, that no useful arrangement has yet been agreed upon. They all arise from a spot upon the under surface of the corpus striatum, known as the locus perforatus anticus, and extend backwards to the posterior cerebral lobes, and to that prominence lodged in the middle fossa of the skull, which marks the hippocampi majores. About the fourth month of fœtal life are developed, within the range of the cerebral hemispheres, and in intimate connexion with one another, the corpora mammillaria, the fornix, and the hippocampi majores, with their transverse commissural fibres, the lyra. The fornix, and the under surface of the corpus callosum, are united by two delicate layers of white and grey matter, constituting the septum lucidum, and inclosing a space called the fifth ventricle, which may contain fluid under circumstances of disease, or may attain very considerable size from congenital malformation.

The spaces known as the lateral ventricles are bounded below by the fornix, the corpora striata, and the optic thalami, and above by the corpus callosum. Three cornua lead into the inferior, posterior, and middle lobes of the hemispheres : in the posterior cornu we see the eminence known as the hippocampus minor; in the inferior, we see the hippocampus major, with the corpus fimbriatum, or the thin margin of the posterior crus of the fornix. The anterior cornu turns outwards round the blunt end of the corpus striatum; the posterior curves in the opposite direction: the inferior cornu winds round the optic thalamus, and ends at the base of the brain, near the division between the anterior and middle lobes, or that interval, the fissure of Sylvius, which receives the processes of Ingrassias of the sphenoid bone. The third ventricle is the space between the optic thalami, under the fornix, and is bounded below by the tuber cinereum, infundibulum, corpora mammillaria, and locus perforatus posticus; it communicates with the lateral ventricles by a fissure, the foramen of Monro, situated between the anterior crura of the fornix and the optic thalami. The iter e tertio ad quartum ventriculum leads from its posterior extremity under the nates and testes. The two optic thalami are united by a narrow band of white fibres, the posterior commissure; the corpora striata are united by a thicker band of white fibres, immediately in front of the anterior crura of the fornix, the anterior commissure: a fusion of the grey matter of the two optic thalami, the middle or soft commissure, may in most instances be seen in the middle of the third ventricle. The character of this commissure approaches that of the fusion of grey matter connecting the two halves of the chord. The position of the pineal body, connected by its two crura to the white investment of the optic thalami, or to the posterior part of the cerebral hemispheres, is remarkable, inasmuch as throughout the whole vertebrate sub-kingdom, whatever may be the size of the hemispheres, its typical relations remain unchanged. The same remark may be applied to the pituitary body, although it is but rarely lodged in so deep a bony cavity as exists in the human skull. The fissure between the cerebral hemispheres and the corpora quadrigemina is called the great transverse fissure, or the fissure of Bichât, and it is here that the pia mater (I believe not the arachnoid) enters the ventricles. It is bounded above by the corpus callosum; below, by the nates and testes; and it extends outwards on either side to the crura cerebri.

The nerves connected with this segment of the encephalon are the optic. They arise from two tubercles of grey matter, situated upon the posterior extremity of the optic thalami, the corpora geniculata externa and interna, and from the nates: the testes have no connexion with the optic nerves. From this extensive origin two flat bands, the optic tracts, wind round the crura cerebri, and unite at the base of the brain to form the commissure or chiasma. Some white fibres seem to pass into the optic tracts from the crura cerebri, and a layer of grey matter is continued from the anterior extremity of the corpus callosum to the upper surface of the commissure. The optic nerves, diverging from this point of union, pass through the optic foramina, at the root of the processes of Ingrassias, and terminate, as the retina, within the fibrous sense-capsule, known as the globe of the eye.

The bony ring surrounding the most anterior of the primary encephalic ganglia is obscured in man by the great development of the ethmoid cells; in its typical form it has the vomer for the centre or body, the two plates of bones constituting the perpendicular lamella, as the neurapophyses; and the ossa nasi as the spine. The cribriform plate of the ethmoid bone supports the olfactory bulbs which represent the last cranial ganglion. Although the cavity for the reception of the olfactory bulbs is small, and retracted between the orbital plates of the frontal bone in man, yet in other animals—the mole, the sheep, the fox—it lies anterior to the frontal bone, forming a distinct, well-circumscribed space, of no inconsiderable size. The bulbs which lie upon it give off numerous delicate nerves to the nose. The soft chords usually termed olfactory nerves are no nerves at all, but direct prolongations of the cerebral substance; in many animals they are hollow, and open into the lateral ventricles; they are composed of white and of grey matter intermixed, a condition which constitutes the main character of a nervous centre. Each olfactory tract is connected to the cerebral hemispheres by three roots, two white and one grey. The external white root crosses the Sylvian fissure, and terminates in the middle or hippocampal lobe: the internal, also white, passes into the locus perforatus anticus, and joins some of the fibres of the anterior commissure within the corpus striatum. The middle or grey root, superior to the other two in the natural position of the brain, arises from a tubercle of grey matter at the posterior extremity of a deep fissure, in which this three-sided olfactory tract is bound down by arachnoid membrane.

From this general survey of the cerebro-spinal axis, it will be seen that the encephalon, or brain commonly so called, is composed of four highly-developed neural segments, of which the posterior epencephalic presides over the functions of respiration and of digestion; the second, mesencephalic, over the act of deglutition, the movements and the sensation of all parts forming the face; the third, prosencephalic, connected with the manifestation of the intellectual faculties, gives to the animal the sense of sight; and the fourth (rhinencephalic) imparts the sense of smell. These latter are the two senses, observes Cuvier, which exercise the greatest influence on the actions of animals, and which minister not only to the preservation of the individual, but also to the conservation of the species. For although the olfactory sense has no connexion with the procreative impulse in man, it has in most of the lower animals, in whom, indeed, it seems to be the main channel through which the sexual stimulus is conveyed.

> Nonne vides, ut tota tremor pertentet equorum

> Corpora, si tantum notas odor attulit auras?

>

> Georg. lib. iii. 250.

---

